# 2505 Mixed emotions: Health care personnel’s reactions to new accountabilities for health equity

**DOI:** 10.1017/cts.2018.257

**Published:** 2018-11-21

**Authors:** Brooke Cunningham, Windy Fredkove, Alden Lai, Dimpho Orionzi, Jill Marsteller

**Affiliations:** 1 CTSI, University of Minnesota; 2 University of Minnesota School of Nursing; 3 Johns Hopkins Bloomberg School of Public Health

## Abstract

OBJECTIVES/SPECIFIC AIMS: Calls for health care organizations to promote health equity, through reducing health care disparities and addressing the social determinants of health, are growing and disrupt assumptions about equal care and the role of the health care delivery system more generally. This paper uses qualitative data to explore the emotions that health care personnel express as they make sense of the newfound emphasis on equity. To do so, we consider the relationships between social identity, sense of control, emotion, cognition, and action. METHODS/STUDY POPULATION: The principle investigator conducted 21 semistructured interviews with senior leaders and equity team members and 7 focus groups with providers and staff employed at one of Minnesota’s largest health care system. The PI asked respondents to describe recent conversations about equity in their workplaces and to identify barriers and facilitators to addressing equity. Focus group participants were also asked to imagine colleagues’ reactions—“what would they say, think, and feel”—should they be asked to adapt practices to address the social determinants of health, community health, and healthcare disparities. Interviews and focus groups were audiotaped and transcribed. Two coders independently coded each transcript for themes and then compared and reconciled their coding. Reactions to equity work emerged inductively during the coding process. RESULTS/ANTICIPATED RESULTS: Findings suggest that discourses on health equity can disrupt personal and professional identities and trigger a mixture of emotions, including fear, sadness, and excitement. Personnel with broad, or flexible, constructions of their work roles experienced less disruption, and more positive emotions, than those personnel who constructed narrow, or rigid, professional identities. Those who expressed a stronger sense of control also expressed more positive emotions, such as happiness and hope, and were excited about the prospect of greater accountabilities related to equity. Those who doubted the existence of disparities were defensive and pointed to cues such as standardized care protocols and perceptions of colleagues’ professionalism to oppose change. Those who perceived low organizational self-efficacy, due to a lack of time, skills, or knowledge, often expressed frustration and helplessness. Their sensemaking focused on the lack of progress and sought sensegiving about ways to “make it workable.” DISCUSSION/SIGNIFICANCE OF IMPACT: Discussions about equity are new in healthcare and trigger mixed reactions, drawing out provider and staff’s hopes, fears, and anxieties. Variations in emotional reactions may be related to differing perceptions about sense of control over disparities and the social determinants of health. If we want to enlist health care providers, nurses, and managers in efforts to improve health equity, we need to understand these emotions and sensemaking processes.

